# The effect of hospital caseload on perioperative mortality, morbidity and costs in bladder cancer patients undergoing radical cystectomy: results of the German nationwide inpatient data

**DOI:** 10.1007/s00345-023-04742-z

**Published:** 2024-01-10

**Authors:** Nikolaos Pyrgidis, Yannic Volz, Benedikt Ebner, Philipp M. Kazmierczak, Benazir Enzinger, Julian Hermans, Alexander Buchner, Christian Stief, Gerald Bastian Schulz

**Affiliations:** 1 Department of Urology, University Hospital, LMU Munich, Marchioninistraße 15, 81377 Munich, Germany; 2 Department of Radiology, University Hospital, LMU Munich, Munich, Germany

**Keywords:** Radical cystectomy, Bladder cancer, Urothelial carcinoma, Perioperative outcomes, Hospital caseload

## Abstract

**Objectives:**

To determine a data-based optimal annual radical cystectomy (RC) hospital volume threshold and evaluate its clinical significance regarding perioperative mortality, complications, length of hospital stay, and hospital revenues.

**Material and methods:**

We used the German Nationwide inpatient Data, provided by the Research Data Center of the Federal Bureau of Statistics (2005–2020). 95,841 patients undergoing RC were included. Based on ROC analyses, the optimal RC threshold to reduce mortality, ileus, sepsis, transfusion, hospital stay, and costs is 54, 50, 44, 44, 71 and 76 cases/year, respectively. Therefore, we defined an optimal annual hospital threshold of 50 RCs/year, and we also used the threshold of 20 RCs/year proposed by the EAU guidelines to perform multiple patient-level analyses.

**Results:**

28,291 (29.5%) patients were operated in low- (< 20 RC/year), 49,616 (51.8%) in intermediate- (20–49 RC/year), and 17,934 (18.7%) in high-volume (≥ 50 RC/year) centers. After adjusting for major risk factors, high-volume centers were associated with lower inpatient mortality (OR 0.72, 95% CI 0.64–0.8, p < 0.001), shorter length of hospital stay (2.7 days, 95% CI 2.4–2.9, p < 0.001) and lower costs (457 Euros, 95% CI 207–707, p < 0.001) compared to low-volume centers. Patients operated in low-volume centers developed more perioperative complications such as transfusion, sepsis, and ileus.

**Conclusions:**

Centralization of RC not only improves inpatient morbidity and mortality but also reduces hospital stay and costs. We propose a threshold of 50 RCs/year for optimal outcomes.

**Supplementary Information:**

The online version contains supplementary material available at 10.1007/s00345-023-04742-z.

## Introduction

Radical cystectomy (RC) with neoadjuvant chemotherapy remains the standard treatment for patients with muscle-invasive or very high-risk non-muscle-invasive bladder cancer. Despite surgical advancements and improvement of inpatient management, RC is still associated with high rates of perioperative morbidity and mortality, prolonged hospital stay and significant treatment-related costs [1, 2]. Perioperative complications occur in more than two-thirds of all patients. Of them, about 20% are severe or life-threatening, prolonging hospital stay and leading to a perioperative mortality of up to 8% [3, 4]. Most common perioperative complications include infections, bleeding and transfusion, sepsis, acute renal failure, ileus, as well as thromboembolism and further cardiopulmonary complications [5]. RC is also associated with considerable perioperative costs, which further increase in case of inpatient complications [6].

Increased annual hospital volume, and not surgeon volume, has been associated with improved perioperative outcomes for major cancer surgeries such as pancreatectomy, esophagectomy and RC [7, 8]. High-volume centers are more likely to provide the required infrastructure and specialized medical and paramedical personnel. Moreover, experienced operative teams can reduce operative time, prevent postoperative complications and optimize the management of intraoperative complications [9]. Therefore, there is an increasing trend toward the centralization of RC [10]. Multiple studies have attempted to identify a hospital volume threshold for annual RC cases that may reduce perioperative morbidity and mortality [11, 12].

Based on the previous notion, the Guideline Panel of the EAU recommends that hospitals should annually perform at least ten, and preferably more than twenty RCs, or otherwise refer patients to centers that reach these numbers [13]. Nevertheless, this recommendation is based on low levels of evidence deriving from a systematic review which included 39 studies. These included studies recruited a relatively low number of participants and reported conflicting data, defining the optimal hospital volume for RC between eight and 55 cases/year [14]. Within this scope, we assessed the GeRmAn Nationwide inpatient Data (GRAND) in order to evaluate the recommended threshold of the EAU and objectively define an optimal annual hospital volume through the largest study on the field.

## Methods

### Data source

Data were retrieved from the Federal Bureau of Statistics of Germany. We assessed inpatient outcomes in Germany from 2005 to 2020. After 2004, all hospitals have to code patient data on previous and inpatient diagnoses, perioperative outcomes, surgeries, and other procedures/interventions. These diagnoses and perioperative outcomes are coded based on the International Statistical Classification of Diseases and Related Health Problems, 10th revision, with German modification (ICD-10-GM), whereas surgeries and procedures/interventions are coded based on the German procedure classification (OPS). These data are mandatory for all German hospitals to get their remuneration and are consequently transferred to the Institute for the Hospital Remuneration System. They are made available for analysis upon request (agreement: LMU-4710-2022) through the Research Data Center of the Federal Bureau of Statistics.

### Selection criteria, coding, and annual hospital caseload threshold

For this study, we included all patients undergoing RC (OPS code: 5-57) due to urinary bladder cancer (ICD code: C67 and D41.4). Patients undergoing concomitant nephroureterectomy were excluded. To obtain information for further procedures, coexisting conditions, and complications, we used the available diagnostic and procedural codes (ICD-10-GM and OPS codes).

The primary outcome of the present study was to define the annual hospital volume threshold that optimizes inpatient outcomes in patients undergoing RC. To identify this optimal threshold, we used the Youden's index based on the receiver operating characteristic (ROC) analysis. The Youden's index was calculated for each point of the ROC curve and its maximum value was selected as a criterion to estimate the optimal threshold for annual hospital caseload and mortality, important complications, length of hospital stay, as well as hospital revenues. Accordingly, the specificity, sensitivity, positive predictive value, and negative predictive value of the annual hospital caseload threshold in optimizing each outcome were also provided.

The optimal annual hospital RC threshold to reduce in-hospital mortality was estimated at 54 cases/year and displayed a sensitivity of 58%, specificity of 48%, positive predictive value of 50%, and negative predictive value of 96%. The optimal annual hospital RC threshold for postoperative ileus was 50 cases and for both sepsis and transfusion 44 cases. Accordingly, the optimal annual hospital RC threshold to reduce the length of hospital stay and the in-hospital costs was 71 and 76 cases, respectively (Data Supplement 1). Therefore, we set an optimal threshold of 50 cases/year to optimize inpatient outcomes in patients undergoing RC. Subsequently, we compared our threshold with the threshold proposed by the current EAU recommendations (≥ 20 cases/year) in terms of inpatient morbidity, mortality, and treatment-related costs.

### Data synthesis and statistical analysis

All hospitals performing RCs were identified through their postal code and were further subclassified based on their annual caseload to low-volume centers (< 20 cases/year), intermediate-volume centers fulfilling the EAU recommendation (20–49 cases/year), and high-volume centers fulfilling the optimal threshold proposed by our analyses (≥ 50 cases/year). The corresponding comparisons among low- (< 20 cases/year) versus intermediate- (20–49 cases/year) versus high-volume centers (≥ 50 cases/year) were performed with the chi-squared and the ANOVA test. All continuous variables were calculated as mean ± standard deviation (SD) and all categorical variables as frequencies with proportions.

A multivariable logistic and linear regression analysis was also performed to assess the role of the recommended from the EAU annual hospital caseload and the optimal annual hospital caseload proposed by our analyses on perioperative mortality and important complications (sepsis, transfusion, and ileus), as well as on the length of hospital stay and hospital revenues. Based on clinical relevance, all models were adjusted for sex, age, obesity, history of chronic obstructive pulmonary disease, heart failure, myocardial infarction, chronic renal failure, cerebrovascular accident, hypertension, diabetes, as well as perioperative acute renal failure, acute respiratory failure, pneumonia, surgical wound infection, and VAC placement. Odds ratios (ORs) with confidence intervals (CIs) were estimated for all logistic models and two-sided p-values ≤ 0.05 were considered statistically significant.

All statistics were performed from the Research Data Center of the Federal Bureau of Statistics based on the R codes supplied by our research team (source: RDC of the Federal Statistical Office and the Statistical Offices of the federal states, DRG Statistics 2005–2020, own calculations).

## Results

### Baseline characteristics

We included a total of 95,841 patients with a mean age of 69 ± 9.8 years. The mean length of hospital stay was 24 ± 14 days, and the in-hospital costs were 17,580 ± 13,110 Euros per patient. A total of 28,291 (29.5%) RCs were undertaken in low-, 49,616 (51.8%) in intermediate- and 17,934 (18.7%) in high-volume centers. 76,190 (79%) patients were men, 8433 (8.8%) were obese, while 53,281 (56%) had hypertension and 18,285 (19%) diabetes. More than half of all patients underwent ileal conduit diversion (52,430, 55%), while a neobladder reconstruction was the second most common urinary diversion (29,700, 31%). A total of 4134 (4.3%) patients underwent a minimal invasive RC. Acute kidney disease occurred in 10,496 (11%) patients, ileus in 9622 (10%), and sepsis in 5429 (5.7%) patients, whereas 50,238 (52%) patients required inpatient transfusion and 4691 (4.9%) VAC placement (Table 1).

**Table 1:** Baseline characteristics of the included patients based on the recommended annual hospital caseload for radical cystectomy

Characteristic	Overall, n = 95,841	< 20, n = 28,291	20–49, n = 49,616	≥ 50, n = 17,934	p-value
Males	76,190 (79%)	22,233 (79%)	39,508 (80%)	14,449 (81%)	** < 0.001**
Age (years)	69 ± 9.8	69 ± 9.6	69 ± 9.8	69 ± 10.2	** < 0.001**
Obesity	8433 (8.8%)	2578 (9.1%)	4464 (9%)	1391 (7.8%)	** < 0.001**
Hypertension	53,281 (56%)	16,056 (57%)	27,583 (56%)	9642 (54%)	** < 0.001**
Diabetes	18,285 (19%)	5594 (20%)	9452 (19%)	3239 (18%)	** < 0.001**
Dementia	1306 (1.4%)	417 (1.5%)	702 (1.4%)	187 (1%)	** < 0.001**
Chronic kidney disease	14,863 (16%)	4796 (17%)	7963 (16%)	2104 (12%)	** < 0.001**
Chronic heart failure	7749 (8.1%)	2574 (9.1%)	4097 (8.3%)	1078 (6%)	** < 0.001**
Chronic cerebrovascular disease	3182 (3.3%)	934 (3.3%)	1750 (3.5%)	498 (2.8%)	** < 0.001**
History of myocardial infarction	1242 (1.3%)	365 (1.3%)	669 (1.3%)	208 (1.2%)	0.16
History of thromboembolism	4131 (4.3%)	1153 (4.1%)	2,023 (4.1%)	955 (5.3%)	** < 0.001**
Chronic obstructive pulmonary disease	11,186 (12%)	3389 (12%)	5939 (12%)	1858 (10%)	** < 0.001**
Hospital stay (days)	24 ± 14	26 ± 15	24 ± 15	22 ± 13	** < 0.001**
Perioperative costs (Euros)	17,580 ± 13,110	17,778 ± 13,350	17,593 ± 13,527	17,279 ± 11,580	**0.003**
Operative technique					** < 0.001**
Open	91,707 (95%)	27,624 (98%)	46,926 (94%)	17,157 (95%)	
Laparoscopic	1,506 (2%)	402 (1%)	963 (2%)	141 (2%)	
Robotic	2,628 (3%)	265 (1%)	1,727 (4%)	636 (4%)	
Ureterocutaneostomy	10,568 (11%)	3524 (12%)	5655 (11%)	1389 (7.7%)	** < 0.001**
Ileal conduit	52,430 (55%)	14,975 (53%)	27,630 (56%)	9825 (55%)	** < 0.001**
Neobladder reconstruction	29,700 (31%)	8574 (30%)	14,823 (30%)	6303 (35%)	** < 0.001**
Colostomy	1667 (1.7%)	478 (1.7%)	902 (1.8%)	287 (1.6%)	0.12
Acute respiratory failure	10,771 (11%)	3,638 (13%)	5,677 (11%)	1,456 (8.1%)	** < 0.001**
Acute kidney disease	10,496 (11%)	3094 (11%)	5543 (11%)	1859 (10%)	**0.012**
Inpatient transfusion	50,238 (52%)	15,636 (55%)	25,263 (51%)	9339 (52%)	** < 0.001**
Inpatient pneumonia	5273 (5.5%)	1665 (5.9%)	2708 (5.5%)	900 (5.0%)	** < 0.001**
Inpatient VAC placement	4691 (4.9%)	1489 (5.3%)	2484 (5.0%)	718 (4.0%)	** < 0.001**
Inpatient sepsis	5429 (5.7%)	1760 (6.2%)	2744 (5.5%)	925 (5.2%)	** < 0.001**
Inpatient ileus	9622 (10%)	2997 (11%)	5020 (10%)	1605 (8.9%)	** < 0.001**

Variables are presented as mean ± standard deviation or frequencies with proportions. The one-way ANOVA test was performed for comparisons among continuous variables and the Chi-squared test for categorical variables. The bold cells indicate statistically significant p-values

### Association of preoperative clinical variables with operative annual volume

**Fig. 1:**
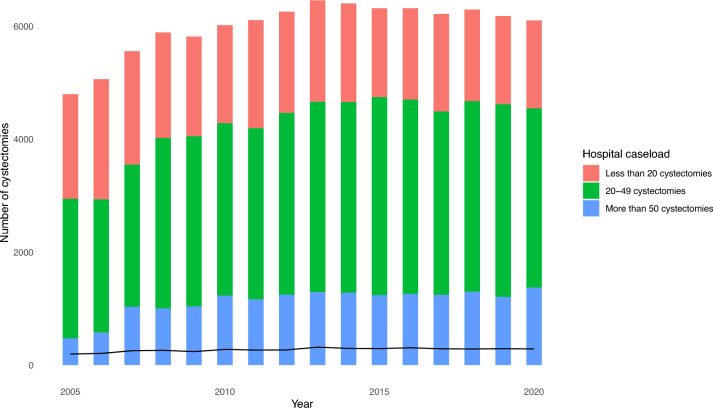
The annual trends for radical cystectomy based on hospital caseload. The line represents the annual in-hospital mortality for all patients

Patients operated in low-volume centers were older and presented a non-clinically significant, slightly higher proportion of hypertension, diabetes, chronic kidney disease, and chronic heart failure compared to intermediate- and high-volume centers. This might be explained by the fact that minimally invasive techniques (p < 0.001) and more complex urinary diversions such as neobladder (p < 0.001) were more common in high-volume centers. Nevertheless, patients operated in high-volume centers displayed fewer postoperative complications. More specifically, the proportion of patients with acute respiratory failure (p < 0.001), acute kidney disease (p = 0.012), pneumonia (p < 0.001), and VAC placement (p < 0.001) was lower in high-volume centers (Table 1). Importantly, both mortality and the annual number of RC remained relative stable throughout the years and were not affected by the COVID-19 pandemic (Fig. 1).

### Effect of hospital caseload on mortality, morbidity, hospital stay, and costs

A total of 4313 (4.5%) patients died after RC during the hospital stay. Of them, 1477 (5.2%) deaths occurred in low-, 2216 (4.5%) in intermediate- and 620 (3.5%) in high-volume centers (p < 0.001). After adjusting for major baseline and perioperative risk factors, intermediate and high-volume centers were associated with a lower inpatient mortality rate compared to low-volume centers (OR 0.84, 95% CI 0.78–0.91, p < 0.001 and OR 0.72, 95% CI 0.64–0.8, p < 0.001, respectively).

Inpatient sepsis, ileus and transfusion were more common in low-volume centers (p < 0.001). After adjusting for major baseline and perioperative risk factors, intermediate- and high-volume centers were associated with lower rates of sepsis compared to low-volume centers (OR 0.89, 95% CI 0.84–0.96, p < 0.001 and OR 0.91, 95% CI 0.83–0.99, p = 0.036, respectively). In the multivariable analysis, the rates of inpatient transfusion were only statistically significant for intermediate-volume centers (p < 0.001), whereas the rates of inpatient ileus were only statistically significant for high-volume centers (p < 0.001) compared to low-volume centers.

Patients undergoing RC in low-volume centers were associated with longer length of hospital stay and more inpatient costs (p < 0.001). After adjusting for major baseline and perioperative risk factors, the length of hospital stay remained shorter by 2.7 (95% CI 2.4–2.9, p < 0.001) days and the inpatient costs were lower by 457 (95% CI 207–707, p < 0.001) Euros in patients undergoing RC in high-volume centers compared to low-volume centers. Patients operated in intermediate-volume centers displayed only statistically significant shorter length of hospital stay by 1.1 (95% CI 0.9–1.3, p < 0.001) days compared to low-volume centers (Supplementary Material 2).

To further evaluate the proposed threshold of 50 RC/year, we compared the outcomes in hospitals performing more than 50 RC/year versus fewer than 50 RC/year (Supplementary Material 3). In the multivariable logistic and linear regression analysis comparing hospitals performing more than 50 RC/year versus fewer than 50 RC/year (Supplementary Material 4), the optimal annual hospital caseload proposed by our analyses was associated with lower rates of mortality (p < 0.001) and inpatient ileus (p < 0.001), as well as with lower length of hospital stay (p < 0.001) and inpatient costs (p = 0.002). In sum, patients undergoing surgery in high-volume centers displayed a 20% decrease in mortality, a 11% decrease in the development of inpatient ileus and stayed 2 days less in hospital after adjusting for major risk factors.

## Discussion

The present high-volume study suggests that the optimal hospital caseload threshold of fifty RCs per year is a major determinant for perioperative morbidity and mortality. Accordingly, this threshold leads to a shorter length of hospital stay and to lower treatment-related costs. Based on multiple analyses and on large-scale data, we demonstrate that increasing the RC volume criteria beyond 20 cases/year to 50 cases/year could optimize inpatient outcomes. Nevertheless, the majority of hospitals in Germany achieved the minimum requirement of 20 RCs per year. However, only 18.7% of patients were operated in high-volume hospitals with more than 50 RC. More specifically, after adjusting for major risk factors, RC in centers achieving 50 cases/year was associated with a 28% reduction in mortality compared to centers performing fewer than 20 cases/year, whereas RC in centers with 20–49 cases/year was associated with a 16% reduction in mortality compared to centers performing fewer than 20 cases/year. Importantly, patients undergoing RC in centers that performed fewer than 20 RC/year developed about 10% more inpatient complications, showcasing that a minimum of 20 RC per year and hospital is required in accordance with the EAU guidelines.

Our findings support the implementation of a minimum hospital caseload threshold for RC. However, based on our data, we propose to increase this threshold at 50 RC/year for optimal perioperative outcomes. In particular, the threshold of 20 RC/year proposed by the Guideline Panel of the EAU was not based on a hospital- or patient-level analysis. Even though the panel performed a systematic review, the low level of available evidence did not permit a direct calculation of a hospital caseload threshold from the existing studies. Therefore, the panel proposed, based on expert opinions, that hospitals should perform at least ten, and preferably more than twenty RCs per year. Based on the previous notion, we demonstrate that hospitals should perform at least twenty, and preferably more than fifty RCs/year. These proposed cutoffs not only lead to substantial improvement in mortality and morbidity but also reduce the length of hospital stay and costs.

Estimations of an optimal hospital threshold for RCs have been previously attempted in the literature. In a retrospective analysis of 6,790 patients, Arora et al. assessed the US National Inpatient Sample, suggesting that major inpatient complications after RC reach a plateau at 45–50 cases/year [14]. Accordingly, Richters et al., employed the Netherlands Cancer Registry including 9,287 patients. This study demonstrated a significant decrease in postoperative mortality between hospitals with more and fewer than 30 RCs [15]. However, most available studies have arbitrarily defined an annual RC caseload from the distribution of hospitals in percentiles based on the data from each database [16–18]. Thus, hospital caseload threshold estimations display significant variety, since hospitals that perform at least two cases/year have been previously defined as low-volume centers, while hospitals that perform more than 50 cases/year have been defined as high-volume centers [19]. To complicate things further, policymakers in countries like the Netherlands or the UK obligate hospitals to perform a minimum of 20 RCs/year or 50 RCs and radical prostatectomies per year [20]. In an attempt to harmonize clinical outcomes and heterogeneity, we suggest that hospitals that perform fewer than 20 RCs/year should refer all patients, whereas hospitals that reach 50 cases/year should be considered referral centers.

Based on our findings, it seems that patients undergoing RC are relatively old with multiple comorbidities [21, 22]. In Germany, continent urinary diversions such as neobladder are performed in about one-third of patients, predominantly in high-volume centers, while minimally invasive techniques are not yet widely implemented, even though they lead to lower transfusion rates [23, 24]. Furthermore, even though the rates of perioperative mortality and morbidity were generally low, acute renal failure and transfusion were among the commonest postoperative complications [25, 26]. Importantly, it seems that, in Germany, most patients display a prolonged hospital stay, which is explained by the fact that the German healthcare insurances cover the perioperative costs. Therefore, clinicians prefer to prolong the patients’ length hospital stay to ensure optimal perioperative management [19, 23]. Interestingly, mortality remained stable throughout the years and the COVID-19 pandemic did not seem to impact the annual number of RCs performed in Germany [27].

Although we provide, to our knowledge, the largest study exploring the annual hospital caseload on perioperative mortality, morbidity, length of hospital stay, and hospital revenues in patients undergoing RC, it should be recognized that our findings were tempered by several limitations. Importantly, our results are based on administrative data, and, therefore, are prone to coding misclassification or inconsistencies. Based on the previous notion, we restricted our morbidity analyses only on sepsis, transfusion and ileus. Similarly, the Clavien-Dindo classification could not be applied. Even though the billing data of the German nationwide inpatient database present a high degree of accuracy, the oncological status of the patients based on their histology, TNM classification, and surgical margins, as well as their laboratory findings are not provided [28]. Accordingly, no data on the annual surgeon’s caseload could be retrieved. Nevertheless, for surgeon volume, only limited and conflicting data exist, suggesting that hospital volume is the main driver of perioperative outcomes [29]. It should be also acknowledged that information on morbidity and mortality after hospital discharge, readmission rates, functional outcomes, recurrence rates, as well as follow-up data are not included in the GRAND study, limiting the extrapolation of our findings. Of note, the GRAND study does not also include data on perioperative medication and fast-track treatments. Therefore, no information about the adherence to antimicrobial prophylaxis or the ERAS protocol could be provided. Still, in an attempt to overcome these limitations, we assessed the role of the hospital caseload of RCs in a holistic approach.

## Conclusion

The present high-volume, real-world data demonstrate that the annual hospital caseload of RCs is a major driver of morbidity, mortality, length of hospital stay, and hospital revenues. Based on our analyses, hospitals that perform at least 50 annual RCs should be considered referral centers. Similarly, given that patients operated in low-volume hospitals develop more postoperative complications, low-volume hospitals should refer patients to centers that reach the minimum requirement of at least twenty RCs per year. Overall, the GRAND study showcases the potential benefits of bladder cancer care centralization not only for patients and hospitals but also for healthcare policymakers in an attempt to reduce length of hospital stay and inpatient costs.

## Supplementary Information

Below is the link to the electronic supplementary material.

Supplementary file1 (DOCX 38 KB)
