# Supplementary material for: The effect of hospital caseload on perioperative mortality, morbidity and costs in bladder cancer patients undergoing radical cystectomy: results of the German nationwide inpatient data
Source: World J Urol. 2024 Jan 10;42(1):19. doi: 10.1007/s00345-023-04742-z (PMC10781819; doi:10.1007/s00345-023-04742-z)
Supplement: Supplementary file 1 — Supplementary file1 (DOCX 38 KB) [file 345_2023_4742_MOESM1_ESM.docx]

Supplementary Material

Table of Contents

[Supplementary Material 1: Youden’s index to define the hospital volume threshold that improves outcomes 2](#_Toc146093557)

[Supplementary Material 2: Regression analysis based on caseload volume of <20, 20-49 and ≥50 RC/year 3](#_Toc146093558)

[Supplementary Material 3: Baseline characteristics based on caseload volume of <20, and ≥50 RC/year 4](#_Toc146093559)

[Supplementary Material 4: Regression analysis based on caseload volume of <50 and ≥50 RC/year 6](#_Toc146093560)

# Supplementary Material 1: Youden’s index to define the hospital volume threshold that improves outcomes

| **Complication** | **Hospital caseload threshold** | **Specificity** | **Sensitivity** | **NPV** | **PPV** |
| --- | --- | --- | --- | --- | --- |
| **Mortality** | 54 | 48% | 58% | 96% | 50% |
| **Sepsis** | 44 | 68% | 35% | 95% | 62% |
| **Transfusion** | 44 | 70% | 34% | 49% | 55% |
| **Ileus** | 50 | 21% | 99% | 91% | 10% |
| **Hospital stay** | 71 | 79% | 58% | 46% | 86% |
| **Costs** | 76 | 71% | 50% | 66% | 50% |

Supplementary Material 1: Annual hospital cystectomy caseload threshold for important perioperative complications after radical cystectomy, based on the maximum Youden's index of the ROC analysis. NPV: negative predictive value, PPV: positive predictive value.

# Supplementary Material 2: Regression analysis based on caseload volume of <20, 20-49 and ≥50 RC/year

| **Annual cystectomy caseload** | **Mortality** | | **Sepsis** | | **Transfusion** | | **Ileus** | | **Length of hospital stay** | | **Costs** | |
| --- | --- | --- | --- | --- | --- | --- | --- | --- | --- | --- | --- | --- |
|  | **OR** | **p-value** | **OR** | **p-value** | **OR** | **p-value** | **OR** | **p-value** | **Beta** | **p-value** | **Beta** | **p-value** |
| <20 | — | — | — | — | — | — | — | — | — | — | — | — |
| 20-49 | 0.84 (0.78, 0.91) | **<0.001** | 0.89 (0.84, 0.96) | **<0.001** | 0.84 (0.82, 0.87) | **<0.001** | 0.96 (0.91, 1.01) | 0.088 | -1.1 (-1.3, -0.92) | **<0.001** | 184 (-16, 383) | 0.071 |
| ≥50 | 0.72 (0.64, 0.8) | **<0.001** | 0.91 (0.83, 0.99) | **0.036** | 0.97 (0.93, 1.01) | 0.09 | 0.87 (0.81, 0.93) | **<0.001** | -2.7 (-2.9, -2.4) | **<0.001** | 457 (207, 707) | **<0.001** |

Supplementary Material 2: Multivariable logistic and linear regression analysis for the effect of annual hospital cystectomy caseload on perioperative mortality, sepsis, transfusion, ileus, length of hospital stay and costs based on the recommended from the EAU annual hospital caseload and on the optimal annual hospital caseload proposed by our analyses. All models are adjusted for sex, age, obesity, history of chronic obstructive pulmonary disease, heart failure, chronic renal failure, cerebrovascular accident, hypertension, diabetes, as well as perioperative acute renal failure, acute respiratory failure, pneumonia, myocardial infarction, surgical wound infection, and VAC placement. The bold cells indicate statistically significant p-values. OR: odds ratio.

# Supplementary Material 3: Baseline characteristics based on caseload volume of <20, and ≥50 RC/year

| **Characteristic** | **<50**, n = 77,907 | **≥50**, n = 17,934 | **p-value** |
| --- | --- | --- | --- |
| **Males** | 61,741 (79%) | 14,449 (81%) | **<0.001** |
| **Age (years)** | 69.3 ± 9.7 | 69 ± 10.2 | **<0.001** |
| **Obesity** | 7,042 (9.0%) | 1,391 (7.8%) | **<0.001** |
| **Hypertension** | 43,639 (56%) | 9,642 (54%) | **<0.001** |
| **Diabetes** | 15,046 (19%) | 3,239 (18%) | **<0.001** |
| **Dementia** | 1,119 (1.4%) | 187 (1%) | **<0.001** |
| **Chronic kidney disease** | 12,759 (16%) | 2,104 (12%) | **<0.001** |
| **Chronic heart failure** | 6,671 (8.6%) | 1,078 (6%) | **<0.001** |
| **Chronic cerebrovascular disease** | 2,684 (3.4%) | 498 (2.8%) | **<0.001** |
| **History of myocardial infarction** | 1,034 (1.3%) | 208 (1.2%) | 0.08 |
| **History of thromboembolism** | 3,176 (4.1%) | 955 (5.3%) | **<0.001** |
| **Chronic obstructive pulmonary disease** | 9,328 (12%) | 1,858 (10%) | **<0.001** |
| **Hospital stay (days)** | 25 ± 15 | 22 ± 13 | **<0.001** |
| **Perioperative costs (Euros)** | 17,656 ± 13,467 | 17,279 ± 11,580 | **0.003** |
| **Operative technique** |  |  | **<0.001** |
| Open | 74,550 (96%) | 17,157 (95%) |  |
| Laparoscopic | 1,365 (1.8%) | 141 (2%) |  |
| Robotic | 1,992 (2.6%) | 636 (4%) |  |
| **Ureterocutaneostomy** | 9,028 (12%) | 1,389 (7.7%) | **<0.001** |
| **Ileal conduit** | 42,605 (55%) | 9,825 (55%) | 0.82 |
| **Neobladder reconstruction** | 14,823 (30%) | 6,303 (35%) | **<0.001** |
| **Colostomy** | 1,380 (1.8%) | 287 (1.6%) | 0.12 |
| **Acute respiratory failure** | 9,315 (12%) | 1,456 (8.1%) | **<0.001** |
| **Acute kidney disease** | 8,637 (11%) | 1,859 (10%) | **0.006** |
| **Inpatient transfusion** | 40,899 (52%) | 9,339 (52%) | 0.31 |
| **Inpatient pneumonia** | 4,373 (5.6%) | 900 (5.0%) | **0.002** |
| **Inpatient VAC placement** | 3,973 (5.1%) | 718 (4.0%) | **<0.001** |
| **Inpatient sepsis** | 4,504 (5.8%) | 925 (5.2%) | **0.001** |
| **Inpatient ileus** | 8,017 (10%) | 1,605 (8.9%) | **<0.001** |

Supplementary Material 3: Baseline characteristics of the included patients based on the recommended annual hospital caseload for radical cystectomy (<50, and ≥50 RC/year). Variables are presented as mean ± standard deviation or frequencies with proportions. The one-way ANOVA test was performed for comparisons between continuous variables and the chi-squared test for categorical variables. The bold cells indicate statistically significant p-values.

# Supplementary Material 4: Regression analysis based on caseload volume of <50 and ≥50 RC/year

| **Annual cystectomy caseload** | **Mortality** | | **Sepsis** | | **Transfusion** | | **Ileus** | | **Length of hospital stay** | | **Costs** | |
| --- | --- | --- | --- | --- | --- | --- | --- | --- | --- | --- | --- | --- |
|  | **OR** | **p-value** | **OR** | **p-value** | **OR** | **p-value** | **OR** | **p-value** | **Beta** | **p-value** | **Beta** | **p-value** |
| <50 | — | — | — | — | — | — | — | — | — | — | — | — |
| ≥50 | 0.8 (0.72, 0.88) | **<0.001** | 0.98 (0.9, 1.06) | 0.5 | 1.08 (1.04, 1.12) | **<0.001** | 0.89 (0.84, 0.95) | **<0.001** | -2 (-2.2, -1.8) | **<0.001** | 335 (124, 546) | **0.002** |

Supplementary Material 4: Multivariable logistic and linear regression analysis for the effect of annual hospital cystectomy caseload on perioperative mortality, sepsis, transfusion, ileus, length of hospital stay and costs based on the optimal annual hospital caseload proposed by our analyses. All models are adjusted for sex, age, obesity, history of chronic obstructive pulmonary disease, heart failure, chronic renal failure, cerebrovascular accident, hypertension, diabetes, as well as perioperative acute renal failure, acute respiratory failure, pneumonia, myocardial infarction, surgical wound infection, and VAC placement. The bold cells indicate statistically significant p-values. OR: odds ratio.
